# The Interplay between Cyclic AMP, MAPK, and NF-*κ*B Pathways in Response to Proinflammatory Signals in Microglia

**DOI:** 10.1155/2015/308461

**Published:** 2015-02-05

**Authors:** Mousumi Ghosh, Vladimir Aguirre, Khine Wai, Hady Felfly, W. Dalton Dietrich, Damien D. Pearse

**Affiliations:** ^1^The Miami Project to Cure Paralysis, University of Miami Miller School of Medicine, Miami, FL 33136, USA; ^2^Department of Neurological Surgery, University of Miami Miller School of Medicine, Miami, FL 33136, USA; ^3^Department of Neurology, University of Miami Miller School of Medicine, Miami, FL 33136, USA; ^4^Department of Cell Biology, University of Miami Miller School of Medicine, Miami, FL 33136, USA; ^5^The Neuroscience Program, University of Miami Miller School of Medicine, Miami, FL 33136, USA; ^6^The Interdisciplinary Stem Cell Institute, University of Miami Miller School of Medicine, Miami, FL 33136, USA

## Abstract

Cyclic AMP is an important intracellular regulator of microglial cell homeostasis and its negative perturbation through proinflammatory signaling results in microglial cell activation. Though cytokines, TNF-*α* and IL-1*β*, decrease intracellular cyclic AMP, the mechanism by which this occurs is poorly understood. The current study examined which signaling pathways are responsible for decreasing cyclic AMP in microglia following TNF-*α* stimulation and sought to identify the role cyclic AMP plays in regulating these pathways. In EOC2 microglia, TNF-*α* produced a dramatic reduction in cyclic AMP and increased cyclic AMP-dependent PDE activity that could be antagonized by Rolipram, myristoylated-PKI, PD98059, or JSH-23, implicating a role for PDE4, PKA, MEK, and NF-*κ*B in this regulation. Following TNF-*α* there were significant increases in iNOS and COX-2 immunoreactivity, phosphorylated ERK1/2 and NF-*κ*B-p65, I*κ*B degradation, and NF-*κ*B p65 nuclear translocation, which were reduced in the presence of high levels of cyclic AMP, indicating that reductions in cyclic AMP during cytokine stimulation are important for removing its inhibitory action on NF-*κ*B activation and subsequent proinflammatory gene expression. Further elucidation of the signaling crosstalk involved in decreasing cyclic AMP in response to inflammatory signals may provide novel therapeutic targets for modulating microglial cell activation during neurological injury and disease.

## 1. Introduction

Microglia play a central role in innate immune responses following CNS injury and during neurodegenerative conditions [1]. Following perturbations of the CNS, microglial cells become activated, altering their morphology, undergoing proliferation, and rapidly migrating to the site of tissue damage or infection. Activated microglia then produce a diverse array of proinflammatory cytokines, oxidative molecules, and degradative enzymes to kill and phagocytose foreign pathogens as well as to remove injured cells and debris [1–3]. Activated microglia can also secrete growth factors and anti-inflammatory cytokines, which are important for tissue remodeling, angiogenesis, and neurorepair [4–6]. These diverse functions of microglia have thus implicated them both in secondary tissue damage [1] as well as in wound repair [7, 8]. Recent classification of activated microglia and macrophages into two main phenotypes, either classically activated (M1) or alternatively activated (M2), has begun to shed light on how these different roles of microglia may be regulated [5, 9, 10].

Cyclic adenosine monophosphate (cyclic AMP) is a ubiquitous cellular second messenger that controls gene expression through protein kinase A- (PKA-) mediated phosphorylation of the constitutively DNA bound transcription factor, the cyclic AMP response element binding protein (CREB; [11]). In microglia and macrophages, elevation of cyclic AMP through the use of adenylyl cyclase (AC) activators, phosphodiesterase (PDE) inhibitors, synthetic cyclic AMP analogs, or *β*-adrenergic receptor agonists has been shown to inhibit the production of proinflammatory genes [12], such as tumor necrosis factor-*α* (TNF-*α*) and tissue factor 1 (TF1), whose expression is positively regulated by the transcription factor, nuclear factor kappa B (NF-*κ*B; [13–15]).

NF-*κ*B is a critical regulator of proinflammatory genes including cytokines and their receptors, cell adhesion molecules, stress-associated proteins, and inducible enzymes that are involved in microglial cell responses [13]. NF-*κ*B is composed of protein dimers that are part of the Rel family of transcription factors, c-rel, p50/p105 (NF-*κ*B1), p65 (RelA), p52/p100 (NF-*κ*B2), and RelB, which all contain the Rel homology domain (RHD) that is important for DNA binding. The NF-*κ*B proteins are sequestered as inactive complexes with inhibitor I*κ*B proteins and are only released when the I*κ*B protein is phosphorylated, ubiquitinated, and degraded [16], which occurs following cellular stimulation with a variety of inducers, including TNF-*α* [17] and interleukin-1*β* (IL-1*β*) [18]. Degradation of I*κ*B allows NF-*κ*B homo- or heterodimers to translocate to the nucleus, bind* cis*-regulatory elements called* kappaB* sites in target genes, and induce their expression [19]. In addition to NF-*κ*B, mitogen-activated protein kinase pathways involving (1) the p38 MAPKs (*α*, *β*, *γ*, and *δ*), (2) c-Jun amino-terminal kinases (JNKs 1, 2, and 3), and (3) extracellular signal-regulated kinases (ERKs 1 and 2) have also been investigated for their role in innate immune responses through both NF-*κ*B-dependent and NF-*κ*B-independent signaling [20].

Previously we reported that the proinflammatory cytokines, TNF-*α* and IL-1*β*, produce a rapid and profound reduction in cyclic AMP in microglia that is accompanied by increased PDE4 expression and activity [21]. We also demonstrated the involvement of PDE4 in cytokine-induced reductions in cyclic AMP in microglia through PDE4 inhibition with Rolipram or molecular knockdown of* pde4b* with RNA interference. The goal of the current study was to examine how signaling intermediaries of the NF-*κ*B, MAPK, and cyclic AMP-PKA-PDE pathways are altered upon TNF-*α*-induced microglial cell activation during the period of cytokine-mediated decreases in cyclic AMP and increased cyclic AMP-dependent PDE activity [21]. Kinases known to phosphorylate and regulate the activity of PDE4, ERK1/2, and PKA [22, 23] were the primary focus of these investigations. We show that inhibitors of PDE4, NF-*κ*B, MEK, and PKA can inhibit proinflammatory cytokine production, antagonize cyclic AMP-dependent PDE activity, and partially or completely reverse cyclic AMP loss following TNF-*α* stimulation of microglia, highlighting that important crosstalk between these multiple pathways converging upon PDE activity is essential for altering the normal homeostatic state of the microglial cell to a classically activated phenotype.

## 2. Materials and Methods

### 2.1. Reagents

Rolipram was purchased from Sigma-Aldrich, (St. Louis, MO). Recombinant mouse tumor necrosis factor (TNF)-*α* was purchased from R&D Systems (Research & Development Systems, Inc., MN). Cyclic AMP analogues (Dibutyryl cyclic AMP and CPT-2′O cyclic AMP) were obtained from BIOLOG Life Science Institute (Bremen, Germany). JSH-23 (4-methyl-N^1^-(3-phenylpropyl)benzene-1,2-diamine) was obtained from EMD Millipore (Billerica, MA). Myristoylated-PKI amide (14-22 amide) was purchased from Enzo Life Sciences (Farmingdale, NY) and PD98059 was obtained from Cell Signaling Technology (Danvers, MA).

### 2.2. Cell Culture

#### 2.2.1. EOC Microglial Cell Culture

The immortalized EOC2 murine microglial cell line (CRL-2467; ATCC, Manassas, VA) was cultured at 37°C, 5% CO_2_ in Dulbecco's modified Eagle's medium (DMEM; Gibco, Life Technologies Corporation, Grand Island, NY) supplemented with 10% heat-inactivated fetal bovine serum (HyClone, Logan, UT), 20% Ladmac conditioned media (ATCC CRL-2420), and 100 units/mL each of penicillin and streptomycin (Sigma-Aldrich). Prior to experimental use, microglia were seeded on either 100 mm culture dishes (1 × 10^6^ cells/dish for biochemical experiments) or onto 8-well chamber slides (5 × 10^5^ cells/well for immunocytochemistry) and grown to ~70% confluence. Cells were treated with various pharmacological inhibitors to specifically block the activity of each signaling pathway: phosphodiesterase-4 (Rolipram; 10 *μ*M), PKA (myristoylated-PKI; 100 nM), MEK (PD98059; 50 *μ*M), or NF-*κ*B translocation into the nucleus (JSH-23; 25 *μ*M) for 30 min prior to stimulation with TNF-*α* (10 ng/mL). Water or DMSO was used as a vehicle-only control in untreated cultures to an endpoint period of 3 or 24 h. Cells were then either lysed for biochemical experiments or fixed with 4% paraformaldehyde (PFA) for immunocytochemistry.

#### 2.2.2. Assessment of Phagocytic Function

The phagocytic function of microglia subjected to various treatment conditions was evaluated based upon their uptake of phycoerythrin- (PE-) conjugated latex beads (Phagocytosis Assay Kit; Cayman Chemical, Ann Arbor, MI) as per the manufacturer's protocol. Following treatment, cells were incubated with the fluorescently labeled beads at 37°C, 5% CO_2_ for a period of 24 h. After incubation, cells were washed twice with 1XPBS for 2 min to remove excess extracellular beads. Cells were then fixed with 4% PFA for 15 min at room temperature, washed for 10 min with 1XPBS, and subsequently immunostained for phalloidin-488. The PE-conjugated latex beads were visualized using fluorescence microscopy. The percentage of phagocytic cells was quantified in 4 random fields per treatment well and averaged across 3 independent experiments.

### 2.3. Molecular Biology

#### 2.3.1. Quantitative Real-Time PCR of* pde4b* mRNA

Total RNA was extracted from EOC2 microglial cells at 3 hours after treatment with TNF-*α* and the various inhibitors as well as from TNF-*α* only stimulated and untreated controls using the Qiagen RNeasy Mini Kit (Qiagen, Valencia, CA). Next, cDNA was synthesized using the Invitrogen Superscript III First Strand DNA Synthesis Kit (Invitrogen, Grand Island, NY). Gene transcripts were quantified by real-time quantitative PCR (qPCR) using the Maxima SYBR Green qPCR Master Mix (Thermo Scientific, Waltham, MA) in triplicate in 12 *μ*L reaction volumes. The sequence-specific primers, synthesized by Sigma-Aldrich, were as follows:* pde4b2* forward 5′-CGGCAAGCAAACAATGAAGG-3′;* pde4b2* reverse 5′-CTGGCCATAGCCGAGTCTCC-3′;* gapdh* forward 5′-CCACCCACAAGACTGTGGAT-3′;* gapdh* reverse 5′-GGATGCAGGGATGATGTTCT-3′. The* n*-fold differential expression was determined by the comparative cycle threshold (CT) method (2^−ΔΔCT^) and normalized to the* gapdh* endogenous reference gene. Negative control experiments were performed without the addition of template cDNA.

### 2.4. Biochemistry

#### 2.4.1. Quantification of Cyclic AMP Levels

The amount of intracellular cyclic AMP in unstimulated and activated microglia subjected to various treatment and control conditions was measured using the cyclic AMP detection kit (cyclic AMP-Glo Assay; Promega Corporation, Madison, WI) as per the manufacturer's instructions. Briefly, the cells were treated with the various inhibitors for a period of 30 min prior to a challenge with TNF-*α* for 3 h. At that time, the cells were lysed using 1X cell lysis buffer (Cell Signaling Technology Inc.), containing IBMX (500 mM; Tocris, Ellisville, MI) and Rolipram (100 *μ*M; Sigma-Aldrich), and then incubated on ice for 30 min. Total cell lysates were centrifuged for 10 min at 1,000 ×g at 4°C. Supernatants were analyzed for total cyclic AMP levels, which were normalized to the total protein concentration in each sample as determined using the BCA protein assay (Bio-Rad Laboratories). Replicates of 3 per treatment and control groups were used. Cyclic AMP levels were expressed as a percentage of the levels in naïve controls.

#### 2.4.2. Quantification of Cyclic AMP-Dependent PDE Activity

The levels of intracellular cyclic AMP-specific PDE activity in resting and activated microglia, subjected to TNF-*α* treatment with/without various inhibitors for a period of 3 hours, was measured in total microglial cell lysates using a cyclic AMP-PDE-specific PDE-Glo ELISA kit (Promega Corporation) as described previously [21]. Briefly, 25 *μ*g of protein was used per assay with each assay carried out in triplicate as per the manufacturer's instructions. The enzymatic reaction was performed using cyclic AMP as a substrate (1 mM final concentration). The reaction mixture was incubated at 25°C for 30 min and then stopped by the addition of PDE-Glo Termination Buffer containing IBMX at a final concentration of 0.5 mM. As a negative control, heat-inactivated cell lysates were incubated under the same conditions. Total cyclic AMP-PDE specific activity was obtained in pmoles/min/mg protein and then expressed as relative values for the different treatment conditions compared to TNF-*α* stimulated controls.

#### 2.4.3. Western Blotting

Immunoblotting was performed on EOC2 microglial cell whole cell lysates or nuclear fractions to identify and quantify the expression and phosphorylation levels of various target proteins altered upon activation with TNF-*α*. For preparation of whole cell lysates, EOC2 microglia were grown on 100 mm plates, subjected to different experimental conditions, and then incubated for 15 min in 0.5 mL ice-cold lysis buffer (Cell Signaling Technology Inc.), containing pepstatin A (1.25 *μ*g/mL), leupeptin (10 *μ*g/mL), aprotinin (25 *μ*g/mL), benzamidine (1 mM), PMSF (0.5 mM), Na_3_VO_4_ (1 mM), NaF (50 mM), Na_4_P_2_O_7_ (2 mM), and 1X Complete Mini Roche Cocktail Protease Inhibitor (Roche, Indianapolis, IN). The lysates were centrifuged (1,000 ×g, 10 min, 4°C) and the supernatants were assayed for total protein. For the preparation of nuclear protein extracts, a combination of low and high ionic strength buffers was used. Cells were first grown on 100 mm plates, subjected to different experimental conditions, and then washed in 1 mL of ice-cold 1X PBS buffer. Cells were removed by gentle scraping and then centrifuged at 1,000 rpm for 5 min at 4°C. The cell pellet was then resuspended in 250 *μ*L of ice-cold 1X hypotonic buffer consisting of HEPES (10 mM, pH 7.9), MgCl_2_ (1.5 mM), KCl (10 mM), EDTA (0.1 mM), DTT (1 mM), 0.1% Nonidet P40, pepstatin A (1.25 *μ*g/mL), leupeptin (10 *μ*g/mL), aprotinin (25 *μ*g/mL), benzamidine (1 mM), PMSF (1 mM), Na_3_VO_4_ (2 mM), NaF (10 mM), Na_4_P_2_O_7_ (2 mM), and 1X Complete Mini Roche Cocktail Protease Inhibitor (Roche) and then incubated for 30 min on ice. The suspension was centrifuged for 10 min at 1,600 rpm in a microcentrifuge at 4°C. The nuclear pellet was suspended in 50 *μ*L of a hypertonic buffer composed of HEPES (20 mM, pH 7.9), glycerol (25%), NaCl (500 mM), MgCl_2_ (1.5 mM), EDTA (0.2 mM), DTT (1 mM), Na_3_VO_4_ (1 mM), NaF (10 mM), Na_4_P_2_O_7_ (2 mM), and 1X Complete Mini Roche Cocktail Protease Inhibitor and incubated for 30 min on ice followed with centrifugation for 10 min at 14,000 rpm. The supernatant constituting the nuclear fraction was analyzed for protein concentration using the BCA protein assay according to the manufacturer's instructions (Bio-Rad Laboratories). The lysates were normalized to a final concentration of 1 mg/mL and boiled at 100°C for 20 min in Laemmli buffer (Bio-Rad Laboratories). A total of 20 *μ*g of protein was loaded into each well of a polyacrylamide gel that was subjected to denaturing gel electrophoresis and subsequently transferred onto nitrocellulose membranes (Bio-Rad Laboratories) as described elsewhere [24]. Following protein transfer, the membranes were blocked with 3% BSA in Tris buffered saline containing 0.1% Tween (TBST; 50 mM Tris HCl (pH 7.4), 150 mM NaCl, and 0.1% Tween) and then probed with specific primary antibodies of interest. The primary antibodies used in this study for Western blotting included p-p65^Ser536^ (1 : 1,000; Cell Signaling Technology Inc.), IkappaB-*α* (1 : 500; Cell Signaling Technology Inc.), PKAcat*α* (1 : 1000, Santa Cruz Biotechnology Inc.), pPKAcat*α* ^Thr197^ (1 : 1,000; Cell Signaling Technology), pERK1/2^Thr-202/Tyr-204^ (1 : 1,000; Santa Cruz Biotechnology Inc.), COX-2 (1 : 1000; Thermo Scientific, Rockford, IL), p-84 nuclear matrix protein (1 : 1000; GeneTex Inc., Irvine, CA), and *β*-actin (1 : 10,000; Sigma-Aldrich). Specific bands were visualized with HRP-conjugated appropriate secondary antibodies (1 : 5,000; Jackson ImmunoResearch Laboratories, Inc., West Grove, PA) using a chemiluminescence detection kit, Supersignal West PICO (Pierce, Rockford, IL), and developed on film (GeneMate, Blue basic Autorad Film, Bioexpress, UT). The optical density of the bands (arbitrary units) was measured with an Imaging Densitometer (Bio-Rad Laboratories) and the levels of each of the specific bands were normalized to *β*-actin p84 Nuclear Matrix protein within each sample run in the same gel.

#### 2.4.4. Immunocytochemistry

The expression, phosphorylation, and cellular localization of signaling intermediaries in unstimulated or TNF-*α* activated microglia were also examined using immunocytochemistry (ICC). Cells were grown on 8-well Lab-Tek II CC^2^ Chamber Slides (Life Science Products, Frederick, CO) and fixed with 4% paraformaldehyde (PFA) in 0.1 M PBS at specific time points following the different treatment conditions. Fixed cells were rinsed using 1X PBS, permeabilized with 0.1% Triton-X 100 for 10 min at 37°C, and then incubated with 2% BSA in 1X PBS for 1 h to block nonspecific binding. After blocking, fixed cells were incubated with the following primary antibodies: p-p65^Ser536^ (1 : 100; Cell Signaling Technology Inc.), pERK1/2^Thr-202/Tyr-204^ (1 : 100; Santa Cruz Biotechnology, Inc., Dallas, TX), pPKAcat*α* ^Thr197^ (1 : 100; Cell Signaling Technology), cyclic AMP (1 : 200; Abcam, Cambridge, MA), COX-2 (1 : 100, Thermo Scientific), iNOS (1 : 100; Abcam), and ED1 (1 : 200; CD68, AbD Serotec, Raleigh, NC) followed by Alexa-594 or Alexa-488 conjugated goat anti-rabbit or goat anti-mouse secondary antibodies (Life Technologies Corporation) as previously described [25]. The morphology of the microglial cells was demarcated using phalloidin-488 (1 : 200; Life Technologies Corporation, Grand Island, NY) when specific microglial cell markers were not used. Hoechst 33342 (1 : 1,000; Life Technologies Corporation) was added during the incubation step with the secondary antibodies to permit labeling of cell nuclei. Immunostained slides were coverslipped with Vectashield mounting medium (Vector Laboratories, Burlingame, CA) and kept protected from light at 4°C until microscopy was performed.

### 2.5. Imaging and Analysis

#### 2.5.1. Confocal Microscopy

Images were acquired by sequential scanning of the immunostained cells with an Olympus Fluorescence Microscope (Olympus, FluoView FV1000) at laser lines of 405, 488, and 594 nm based on the specific Alexa-fluor secondary antibody tag used. In Adobe Photoshop CS6 (Adobe Systems, San Jose, CA) all fluorescent images had the same universal adjustments applied: brightness (+50), contrast (+30), and smart sharpen (1.0 pixels).

#### 2.5.2. Measurement of Fluorescent Intensity per Cell

The per cell fluorescence intensity of immunostaining for the different markers probed in microglia was quantified using Image J software (http://imagej.nih.gov/). For each treatment condition, 10–15 randomly selected cells identified by Hoechst-positive nuclei were imaged per plate. These images were converted to grayscale and the average per pixel intensity of the signal per cell (arbitrary units, a.u.) was recorded after background subtraction. These measurements were averaged across the cells analyzed per plate and then among group replicates for comparison of signal change across treatments.

### 2.6. Statistics

Significant differences between groups were ascertained by one-way analysis of variance (ANOVA), followed by Bonferroni post hoc analysis using GraphPad Prism 4.0 (GraphPad Software, La Jolla, CA). All data was analyzed at the 95% confidence interval. Graphed data was expressed as the mean ± standard error of the mean (SEM) according to the number of replicates or independent experiments performed. Asterisks or hashes included on the graphs indicate statistical differences between the treatment and control condition(s) with significance indicated at ^∗∗∗/###^ *P* < 0.001, ^∗∗/##^ *P* < 0.01, or ^∗/#^ *P* < 0.05.

## 3. Results

### 3.1. TNF-*α*-Induced Reductions in Cyclic AMP Are Reversed through Inhibition of PDE4, PKA, MEK, or NF-*κ*B Nuclear Translocation

**Figure 1:**
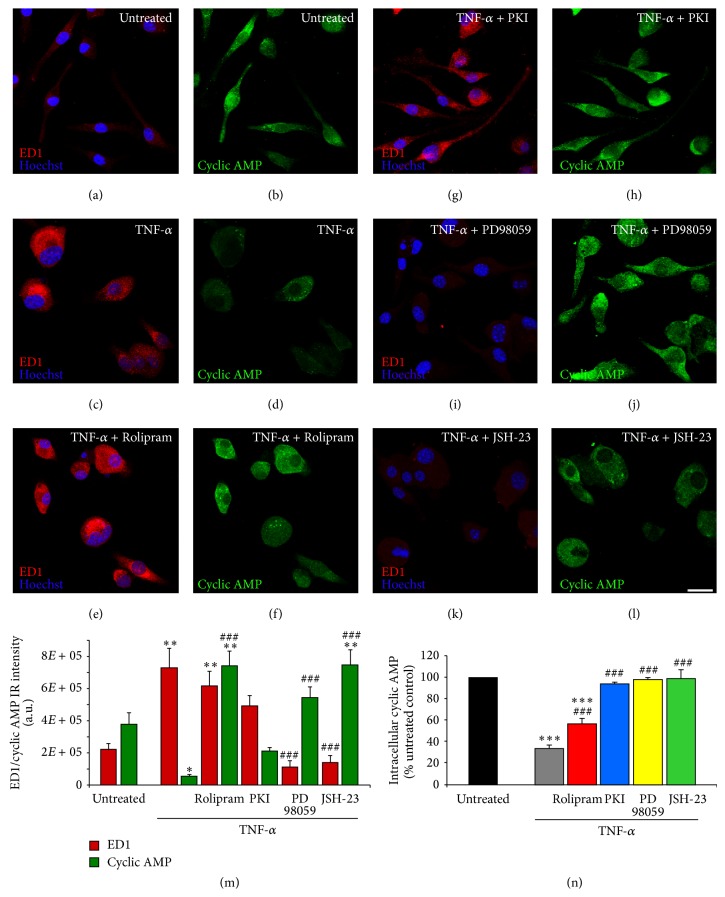
Reduced cyclic AMP levels in EOC2 microglia following TNF-*α* are rescued by PDE4, MEK, m-PKI, or NF-*κ*B p65 inhibition. Unstimulated, resting EOC2 microglia express very little ED1 ((a) red) and exhibit significant intracellular immunoreactivity for cyclic AMP ((b) green). Upon activation with TNF-*α*, there was a marked induction of ED1 (c) and a dramatic reduction in cyclic AMP (d) within 3 h. Inhibiting PDE4 activity with Rolipram in TNF-*α* treated microglial cells did not alter ED1 expression (e) but restored cyclic AMP (f). The antagonism of PKA with m-PKI did not alter the production of either ED1 (g) or cyclic AMP (h). Inhibiting MEK with PD98059 or p-p65^Ser536^ NF-kB nuclear translocation with JSH-23 reduced ED1 ((i) and (k), resp.) and also restored cyclic AMP ((j) and (l), resp.). Scale bar = 20 *μ*m. Measurements of ED1 (red) and cyclic AMP (green) immunoreactive (IR) signal intensity were obtained in microglia using fluorescent microscopy and Image J software for the different imaged conditions (m). Quantitation of cyclic AMP concentrations in TNF-*α* challenged microglial cells (i) showed the restoration of cyclic AMP to resting levels following treatment with inhibitors of PKA, ERK, and NF-*κ*B and partially with PDE4. Results shown are the average from three independent culture plate replicates for each treatment condition examined. Cell nuclei are visualized using the nuclear stain Hoechst (blue). Errors are given as SEMs. Statistical significance relative to naive controls is indicated at ^*^ *P* < 0.05, ^**^ *P* < 0.01, or ^***^ *P* < 0.001 and versus TNF-*α* stimulated cells at ^###^ *P* < 0.001.

Initially, cyclic AMP changes and microglial cell activation, as evidenced by the presence of the lysosomal marker macrosialin (ED1; [26]), were examined in EOC2 microglial cells following stimulation with TNF-*α* (10 ng/mL) using immunocytochemistry with specific antibodies. In naïve microglia, which expressed very little ED1 (Figure 1(a)), cyclic AMP immunoreactivity was pronounced throughout the cytoplasm (Figure 1(b)). Within 3 hours of TNF-*α* stimulation, the microglial cells became activated, as evidenced morphologically with the rounding of the cell body and loss of their original spindle or stellate appearance and robust expression of ED1 (Figure 1(c)). In many of the microglial cells, cyclic AMP immunoreactivity was completely lost or faint in appearance (Figure 1(d)). When TNF-*α* stimulation was combined with the simultaneous addition of the PDE4 inhibitor Rolipram (10 *μ*M), although ED1 staining remained relatively unchanged compared to TNF-*α* stimulation alone (Figure 1(e)), cyclic AMP immunoreactivity was now prominent throughout the cytoplasm of the EOC2 microglia at 3 hours after stimulation (Figure 1(f)). Conversely, the inhibition of PKA with m-PKI (100 nM) did not significantly affect TNF-*α* induced changes in either ED1 (Figure 1(g)) or cyclic AMP immunoreactivity (Figure 1(h)) in this assay. When either the MEK-inhibitor PD98059 (50 *μ*M) or the NF-*κ*B nuclear translocation inhibitor JSH-23 (25 *μ*M) were added simultaneously with TNF-*α*, both the induction of ED1 (Figures 1(i) and 1(k), resp.) and the loss of cyclic AMP immunoreactivity were blocked within 3 hours after stimulation (Figures 1(j) and 1(l), resp.). These experiments were repeated three times and the fluorescent intensity of immunostaining was quantified (Figure 1(m)). The images shown in Figure 1 are representative of the immunocytochemical changes observed with each condition.

To confirm the immunocytochemical findings of cyclic AMP loss in EOC2 microglia after TNF-*α* stimulation (10 ng/mL) quantitatively, cyclic AMP was measured in cell lysates harvested at 3 hours following TNF-*α* treatment (Figure 1(n)). For each condition, replicates of 3 were employed and measurements were normalized to levels of cyclic AMP in naïve microglial cells. At 3 hours after TNF-*α* stimulation, as observed with immunocytochemistry, a pronounced reduction in cyclic AMP occurred (gray bar; reduction to 33.6 ± 1.5% of naïve levels, *P* < 0.001 versus naive). The simultaneous addition of  10 *μ*M Rolipram partially reversed the TNF-*α*-induced reduction in cyclic AMP (red bar; reduction to 56.0 ± 1.8% of naïve levels, *P* < 0.001 versus naïve, and *P* < 0.001 versus TNF-*α*). TNF-*α*-induced reductions in cyclic AMP, however, were completely reversed in the presence of the PKA-inhibitor m-PKI (100 nM; blue bar, 93.7 ± 0.6% of naïve levels, n.s. compared to naïve, and *P* < 0.001 versus TNF-*α*), the MEK-inhibitor PD98059 (50 *μ*M; yellow bar, 97.3 ± 0.7% of naïve levels, n.s. compared to naïve, and *P* < 0.001 versus TNF-*α*), or the NF-*κ*B nuclear translocation inhibitor JSH-23 (25 *μ*M; green bar, 98.6 ± 4.4% of naïve levels, n.s. compared to naïve, and *P* < 0.001 versus TNF-*α*; Figure 1(n)).

### 3.2. TNF-*α*-Stimulated Production of the M1 Proinflammatory Markers COX-2 and iNOS in Microglia Is Perturbed by Antagonism of PDE4 Activity, PKA, MEK, or NF-*κ*B Translocation

**Figure 2:**
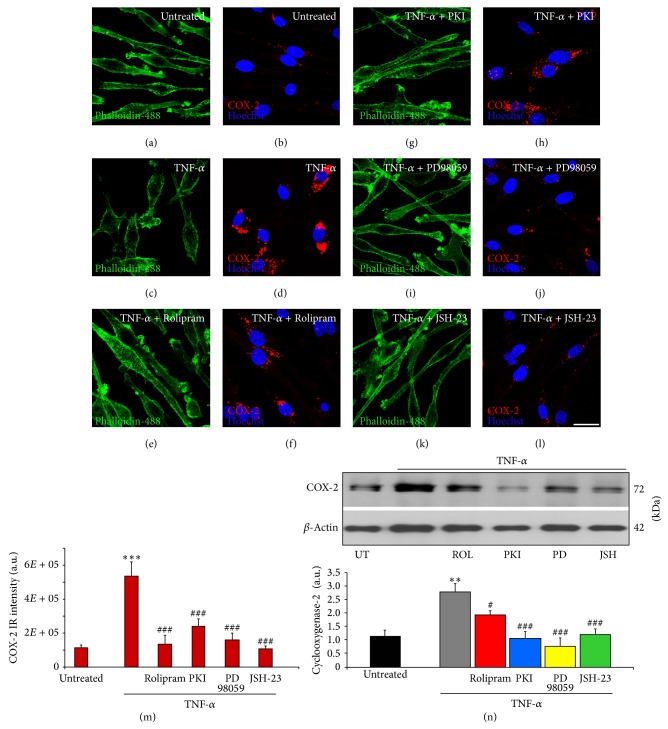
TNF-*α* induced increases in COX-2 are perturbed by the inhibition of PDE4, MEK, PKA, or NF-*κ*B p65 in microglia. Unstimulated, resting EOC2 microglia, the morphology of which was demarcated by phalloidin-488 staining ((a) green), showed very little expression of COX-2 ((b) red). Upon stimulation with TNF-*α*, there was a marked production within 3 h of COX-2 in the cytoplasm (d). Inhibiting PDE4 activity with Rolipram (f), PKA with m-PKI (h), MEK with PD98059 (j), or NF-*κ*B translocation with JSH-23 (l) significantly reduced COX-2 immunoreactivity in TNF-*α* treated microglial cells. Measurements of COX-2 (red) immunoreactive signal intensity were obtained in microglia using fluorescent microscopy and Image J software for the different imaged conditions (m). Confirmation of changes in COX-2 was then confirmed by immunoblot (n). Quantification of COX-2 levels was performed by densitometry analysis (arbitrary units) normalized to *β*-actin. Stimulation of microglia with TNF-*α* (gray bar) produced a significant increase in COX-2 band intensity, recognized at 72 kDa, compared to untreated controls (black bar). Antagonism of PDE4 (red bar), PKA (blue bar), MEK (yellow bar), or NF-*κ*B (green bar) all significantly perturbed the increase in COX-2 observed in microglia after TNF-*α* stimulation. Results shown are the average from three independent culture plate replicates for each treatment examined. Cell nuclei are visualized using the nuclear stain Hoechst (blue). Scale bar = 20 *μ*m. Errors are given as SEMs. Statistical significance relative to naive controls is indicated at ^**^ *P* < 0.01 and versus TNF-*α* stimulated cells at ^#^ *P* < 0.05 or ^###^ *P* < 0.001.

**Figure 3:**
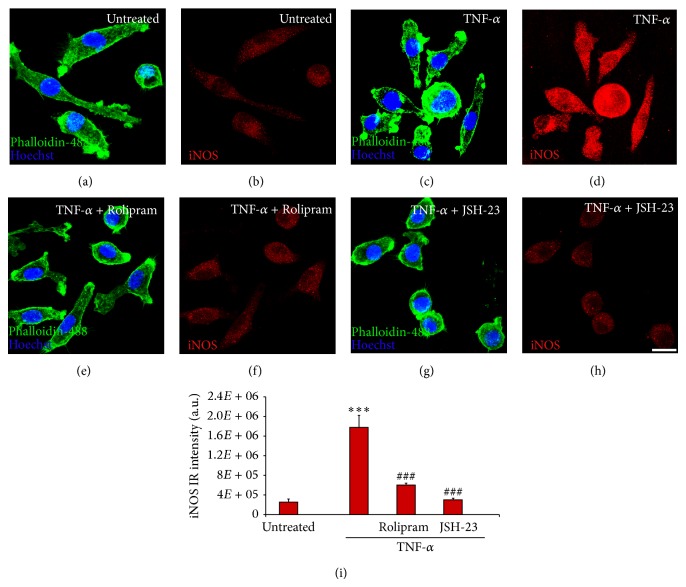
TNF-*α* induced increases in iNOS are inhibited by the antagonism of PDE4 or NF-*κ*B p65 in microglia. In unstimulated, resting EOC2 microglia with their morphology shown by phalloidin-488 staining ((a) green), iNOS expression was low ((b) red). Upon stimulation with TNF-*α* there was significant iNOS production within 3 h (d). Inhibiting PDE4 activity with Rolipram (f) or NF-*κ*B translocation with JSH-23 (h) significantly reduced iNOS immunoreactivity in TNF-*α* treated microglial cells. Measurements of iNOS (red) immunoreactive (IR) signal intensity were obtained in microglia using fluorescent microscopy and Image J software for the different imaged conditions (m). Results shown are the average from three independent culture plate replicates for each treatment examined. Cell nuclei are visualized using the nuclear stain Hoechst (blue). Scale bar = 20 *μ*m. Errors are given as SEMs. Statistical significance relative to naive controls is indicated at ^***^ *P* < 0.001 and versus TNF-*α* stimulated cells at ^###^ *P* < 0.001.

To further examine the effects of blocking specific signaling cascades on microglial cell activation, the characteristic markers of the macrophage-microglia M1 proinflammatory phenotype COX-2 and iNOS were examined biochemically and immunohistochemically in TNF-*α* stimulated microglia. For each condition, replicates of 3 were employed. In untreated, phalloidin-488 costained microglia (Figure 2(a)), little immunoreactivity for COX-2 was evident (Figure 2(b)). At 3 hours after stimulation with TNF-*α*, a marked increase in COX-2 immunoreactivity was observed in the cytoplasm of EOC2 microglia (Figures 2(c)-2(d), 2(m)). Inhibition of PDE4 with Rolipram (Figures 2(e)-2(f)), PKA with m-PKI (Figures 2(g)-2(h)), MEK with PD98059 (Figures 2(i)-2(j)), or NF-*κ*B translocation with JSH-23 (Figures 2(k)-2(l)) all abrogated TNF-*α* induced increases in COX-2 staining as quantified by immunoreactive intensity measurements in microglia (Figure 2(m)). Elevated COX-2 after TNF-*α* stimulation and its reduction upon antagonism of these signaling pathways was further confirmed by immunoblot analysis (Figure 2(n)). Stimulation of microglia with TNF-*α* produced a significant increase in the intensity of the recognized COX-2 band (72 kDa) at 3 hours (242% increase, *P* < 0.001 as compared to untreated controls and normalized to *β*-actin). Significant reductions in COX-2 compared to TNF-*α* only stimulated controls were observed when Rolipram (30.5% reduction, *P* < 0.05), m-PKI (61.6% reduction, *P* < 0.05), PD98059 (71.8% reduction, *P* < 0.05), or JSH-23 (56.4% reduction, *P* < 0.05) were coadministered with TNF-*α* (Figure 2(n)). Similar to COX-2, TNF-*α* stimulation produced a pronounced increase in the immunoreactivity for the proinflammatory marker iNOS at 3 hours (711% increase, *P* < 0.001 compared to untreated controls in Figures 3(a)-3(b)) within EOC2 microglia (Figures 3(c)-3(d)). The inhibition of PDE4 (Figures 3(e)-3(f)) or NF-*κ*B translocation (Figures 3(g)-3(h)) was able to prevent TNF-*α* induced increases in iNOS in microglia (Figure 3(i); *P* < 0.001 compared to TNF-*α* stimulated controls, n.s. compared to untreated controls for both treatments).

### 3.3. TNF-*α* Stimulation of EOC2 Microglial Cells Alters the Degree of PKA and ERK 1/2 Phosphorylation as well as Its Subcellular Localization

**Figure 4:**
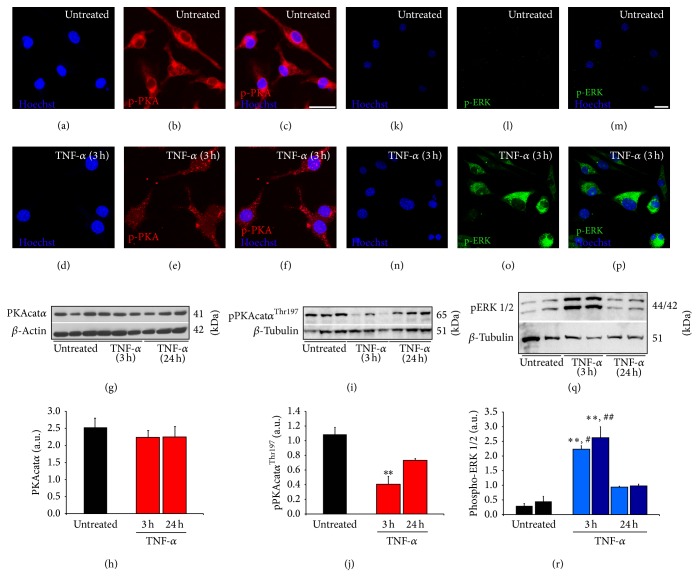
TNF-*α* reduced levels of catalytic pPKAcat*α* ^Thr197^ and altered its subcellular localization while increasing cytosolic levels of activated pERK 1/2^Thr-202/Tyr-204^ in EOC2 microglia. Compared to the levels of pPKAcat*α* ^Thr197^ (red) in resting EOC2 microglia (a–c), TNF-*α* stimulation (d–f) produced a significant reduction in pPKAcat*α* ^Thr197^ immunoreactivity as well as altered its subcellular localization from a diffuse to particulate cytosolic appearance at 3 h. Western blot analysis of the total cell lysates of untreated and TNF-*α* stimulated EOC2 microglia showed no change in total PKAcat*α* (g-h) and confirmed quantitatively the significant reduction in pPKAcat*α* ^Thr197^ within 3 h of stimulation, which remained depressed through 24 h (i-j). Quantification of pPKAcat*α* ^Thr197^ levels using immunoblot densitometry values (arbitrary units) normalized to *β*-actin (j). Conversely, pERK 1/2^Thr-202/Tyr-204^ immunoreactivity (green), which was almost undetectable in resting microglia (k–m), was significantly increased within 3 h of stimulation with TNF-*α* (n–p). Western blot analysis confirmed the upregulation of pERK 1/2^Thr-202/Tyr-204^ at 3 h post-TNF-*α* activation, though by 24 h these levels had returned to near normal values (q-r). Quantification of ERK 1/2^Thr-202/Tyr-204^ levels by densitometry analysis (arbitrary units) normalized to *β*-actin (r). The results shown are the averages from three independent culture plate replicates for each treatment condition; the bands from all three (g) or two (o) of the replicates are presented on the blot images. Scale bars = 20 *μ*m. Errors are given as SEMs. Statistical significance relative to naive controls is indicated at ^**^ *P* < 0.01 and versus 24 h post-TNF-*α* stimulation at ^#^ *P* < 0.05 or ^##^ *P* < 0.01.

Following the identification of signaling pathways involved in TNF-*α*-induced reductions in cyclic AMP in microglial cells, these experiments examined how these pathways were altered upon TNF-*α* challenge. To accomplish this goal, both immunocytochemistry and Western blotting for signaling intermediaries were performed at 3 and 24 hours after TNF-*α* stimulation, using 3 replicates for each condition. For maximal activation, the catalytic subunit of PKA (PKAcat) requires phosphorylation on its Thr-197 residue by itself or PDK-1 [27]. In untreated EOC2 microglia pPKAcat*α* ^Thr197^ was detected throughout the cytoplasm of the cells by immunocytochemistry (Figures 4(a)–4(c)). At 3 hours following TNF-*α* stimulation, there appeared to be less immunoreactivity for pPKAcat*α* ^Thr197^ and although its localization remained cytoplasmic, it was particulate (Figures 4(d)–4(f)), possibly indicating that it was being degraded and/or relocated to a specific subcellular compartment upon TNF-*α* stimulation. Western blotting showed that there was no change in the total amount of PKAcat*α* at both 3 and 24 hours after TNF-*α* stimulation in microglia (Figures 4(g)–4(h); red bars, n.s. change compared to untreated controls), though it confirmed a marked reduction in the amount of pPKAcat*α* ^Thr197^ at 3 hours (red bars; 37.0 ± 10.1% of untreated levels, *P* < 0.01 versus untreated) compared to untreated cells (black bars). Although at 24 hours there were greater amounts of pPKAcat*α* ^Thr197^, they did not appear to be as high as that of untreated controls (Figures 4(i)-4(j)).

Next, we examined changes in the phosphorylation of ERK1/2 at Thr-202/Tyr-204, respectively, which is one of the main downstream kinase targets of the Ras-Raf-MEK-ERK signaling cascade [28]. Using immunocytochemistry, in untreated EOC2 microglia very little p-ERK 1/2^Thr-202/Tyr-204^ was detected (Figures 4(k)–4(m)). At 3 hours following TNF-*α* stimulation, there was a robust immunoreactivity for p-ERK1/2^Thr-202/Tyr-204^ throughout the cytoplasm of the cells (Figures 4(n)–4(p)). Western blotting confirmed the significant increase in p-ERK1/2^Thr-202/Tyr-204^ at 3 hours after TNF-*α* stimulation of microglia (blue bars; 7.8-fold (p-ERK1^Thr-202^) and 5.9-fold (p-ERK2^Tyr-204^) increases over untreated, *P* < 0.01 for both p-ERK1 and 2. At 24 hours, like pPKAcat*α* ^Thr197^, the amount of p-ERK 1/2^Thr-202/Tyr-204^ had returned to near that of untreated controls (Figures 4(q)-4(r)).

### 3.4. TNF-*α* Alters NF-*κ*B p65 Phosphorylation, I*κ*B Degradation, and NF-*κ*B p65 Translocation That Are Perturbed by PDE4 Inhibition or Cyclic AMP Elevation

**Figure 5:**
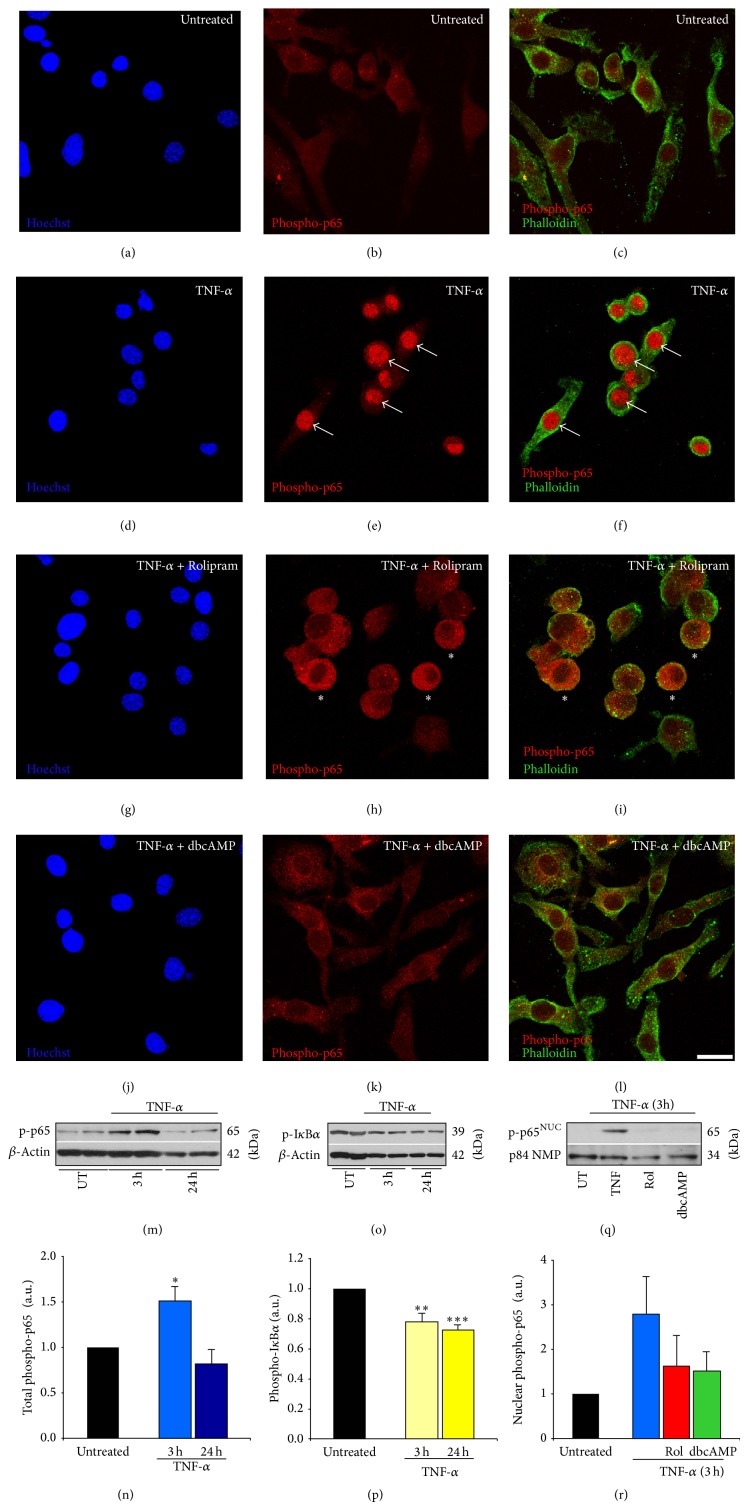
Elevating cyclic AMP levels in TNF-*α* activated microglia using the PDE4 inhibitor Rolipram or a cyclic AMP analog inhibits nuclear translocation of p-p65^Ser-536^. Resting EOC2 microglial cells (a–c) showed very little p-p65^Ser-536^ immunoreactivity (red). Within 3 h of TNF-*α* stimulation, robust nuclear immunoreactivity for p-p65^Ser-536^ was observed (d–f). Treatment of TNF-*α* activated cells with either the PDE4 inhibitor Rolipram (g–i) or the cyclic AMP analogue db-cyclic AMP (j–l) significantly reduced nuclear p-p65^Ser-536^ immunoreactivity, with p-p65^Ser-536^ appearing to have been sequestered in the cytoplasm. Cell morphology is demarcated by staining with Alexa 488-tagged phalloidin (green) and the nuclear dye Hoechst (blue). Scale bar = 30 *μ*m. White arrows and white asterisks show pronounced p-p65^Ser-536^ immunoreactivity within the nuclear and cytoplasmic compartments, respectively, among TNF-*α* stimulated microglia and TNF-*α* stimulated microglia with Rolipram. Immunoblotting of EOC2 microglial cell lysates showed significantly elevated levels of p-p65^Ser-536^ at 3 h following stimulation with TNF-*α* (m), which had returned to normal levels by 24 h. Quantification of p-p65^Ser-536^ levels by densitometry analysis (arbitrary units) normalized to *β*-actin (n). Immunoblotting for p-I*κ*B*α* following TNF-*α* showed a persistent decrease through 24 h in EOC2 microglial cells (o). Quantification of p-I*κ*B*α* levels by densitometry analysis (arbitrary units) normalized to *β*-actin (p). Western blot analysis of the purified nuclear fraction showed a trend towards elevated levels of nuclear p-p65^Ser-536^ following TNF-*α*, which did not occur in the presence of cyclic AMP elevating agents Rolipram or db-cyclic AMP (q). Quantification of nuclear p-p65^Ser-536^ levels by densitometry analysis (arbitrary units) normalized to p-84 nuclear matrix protein (r) revealed a trend for a cyclic AMP effect on nuclear p-p65^Ser-536^ after TNF-*α*, though no statistical significance was indicated. The results shown are the averages from four independent culture plate replicates for each treatment condition; the bands from up to two of the replicates are presented on the blot images. Errors are given as SEMs. Statistical significance to naive controls is indicated at, ^*^ *P* < 0.05,  ^**^ *P* < 0.01, or ^***^ *P* < 0.001.

The NF-*κ*B pathway is the main effector cascade downstream of proinflammatory cytokines in microglia that is responsible for inducing gene expression changes involved in their morphological and immunophenotypical transition to a classically activated phenotype [29]. In these experiments, we investigated how NF-*κ*B signaling was altered by TNF-*α* at the stages of transcription factor (p65) phosphorylation at Ser-536, p-p65^Ser-536^ untethering from the cytoplasm through I*κ*B degradation and its subsequent nuclear translocation, as well as how these processes were altered by increasing cyclic AMP, either through the addition of the PDE4 inhibitor Rolipram or through a synthetic cyclic AMP analog, dibutyryl cyclic AMP. These experiments involved both immunocytochemical and Western blot analyses using replicates of 4 per condition, except for the p-p65^Ser-536^ immunoblot in which 2 replicates were employed. In untreated EOC2 microglia, very little p-p65^Ser-536^ was found throughout the cytoplasm of the cells (Figures 5(a)–5(c)). At 3 hours after TNF-*α* stimulation, there was a significant increase in p-p65^Ser-536^ and it was almost exclusively localized to the nuclear compartment (Figures 5(d)–5(f)). The concurrent addition of either 10 *μ*M Rolipram (Figures 5(g)–5(i)) or 1 mM db-cyclic AMP (Figures 5(j)-5(l)) completely prevented the nuclear translocation of p-p65^Ser-536^ at 3 hours after TNF-*α* stimulation in microglia, with db-cyclic AMP also reducing the apparent total amount of p-p65^Ser-536^. Immunoblotting demonstrated an increase in NF-*κ*B p-p65^Ser-536^ at 3 hours after TNF-*α* stimulation (Figures 5(m)-5(n)), which occurred in the presence of a significant reduction in p-I*κ*B*α* (Figures 5(o)-5(p)), and resulted in its nuclear translocation; cyclic AMP elevation, through the administration of Rolipram or db-cyclic AMP, appeared to antagonize this translocation of p-p65^Ser-536^ (Figures 5(q)-5(r)).

### 3.5. Antagonism of PDE4, PKA, MEK, and NF-*κ*B Reduces Cyclic AMP-Dependent PDE Activity in TNF-*α* Stimulated Microglia

**Figure 6:**
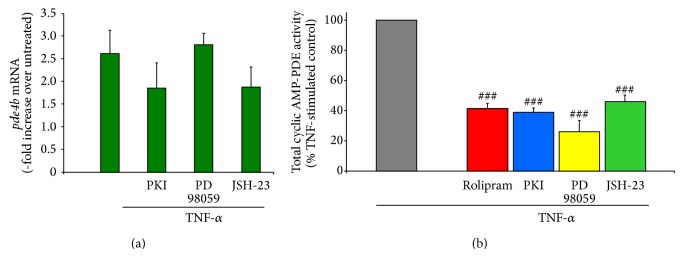
Antagonism of PDE4, PKA, MEK, and NF-*κ*B reduces cyclic AMP-dependent PDE activity in TNF-*α* stimulated microglia. In TNF-*α* stimulated microglia, the effect of antagonism of PKA, MEK, and NF-*κ*B on* pde4b2* expression was measured using Q-PCR (a). At 3 h after exposure to TNF-*α*,* pde4b2* mRNA expression increased 2.5–3.0-fold in microglia compared to untreated controls, similar to that previously reported [21]. The addition of m-PKI or JSH-23, but not PD98059, showed a trend for a reduction in* pde4b2* expression, though it was not statistically significant at this time point. Next, the effect of inhibiting PDE4, PKA, MEK, and NF-*κ*B on cyclic AMP-dependent PDE activity in microglia at 3 h after TNF-*α* challenge was investigated (b). Compared to TNF-*α* only stimulated controls (gray bar), the addition of Rolipram (red bar), m-PKI (blue bar), PD98059 (yellow bar), or JSH-23 (green bar) all significantly reduced cyclic AMP-dependent PDE activity by at least 50%. The results shown are obtained from three independent culture plate replicates for each treatment condition. Errors are given as SEMs. Statistical significance versus TNF-*α* stimulated cells is given at ^###^ *P* < 0.001.

To examine how the antagonism of the different signaling pathways may be involved in preventing microglial cell activation and the production of proinflammatory mediators upon TNF-*α* stimulation, the convergence of these pathways on PDE expression and activity were examined through inhibition experiments. To accomplish this goal, both quantitative real-time PCR and cyclic AMP-dependent PDE activity assays were performed on treated and control microglia at 3 hours after TNF-*α* stimulation using 3 replicates for each condition. Previously, we identified that* pde4b* mRNA and protein are significantly increased upon TNF-*α* stimulation in microglia and that molecular perturbation of* pde4b* expression using short-hairpin RNA could prevent TNF-*α* induced cyclic AMP decreases in microglia [21]. Therefore, we examined if by inhibiting specific signaling pathways in microglia after TNF-*α* stimulation, whether changes in* pde4b* mRNA expression may occur. We found that there was a trend for a reduction in* pde4b* mRNA when either PKA or NF-*κ*B inhibition was employed but not MEK (Figure 6(a)), suggesting that transcriptional activation of* pde4b* gene expression after TNF-*α* stimulation may occur downstream of these signaling pathways to in part decrease cyclic AMP through increased PDE4.

We have also previously shown that TNF-*α* stimulation of microglia produces a marked increase in cyclic AMP-dependent PDE activity that temporally matches the decrease in cyclic AMP that occurs in these cells [21]. In the current study, the application of inhibitors to PDE4, PKA, MEK, and NF-*κ*B, in conjunction with TNF-*α*, all showed an ability to antagonize TNF-*α*-induced cyclic AMP-dependent PDE activity (Figure 6(b); reductions compared to TNF-*α* alone, Rolipram, 41.4 ± 3.4%; m-PKI, 38.8 ± 2.9%; PD98059, 26.0 ± 7.3%; and JSH-23, 45.9 ± 4.3%). The change observed mirrored the effects of these agents on cyclic AMP suggesting that there is a convergence of these pathways upon the regulation of PDE activity which in turn alters levels of intracellular cyclic AMP.

### 3.6. NF-*κ*B and PKA Are Important for TNF-*α*-Induced Increases in Microglial Cell Phagocytic Function

**Figure 7:**
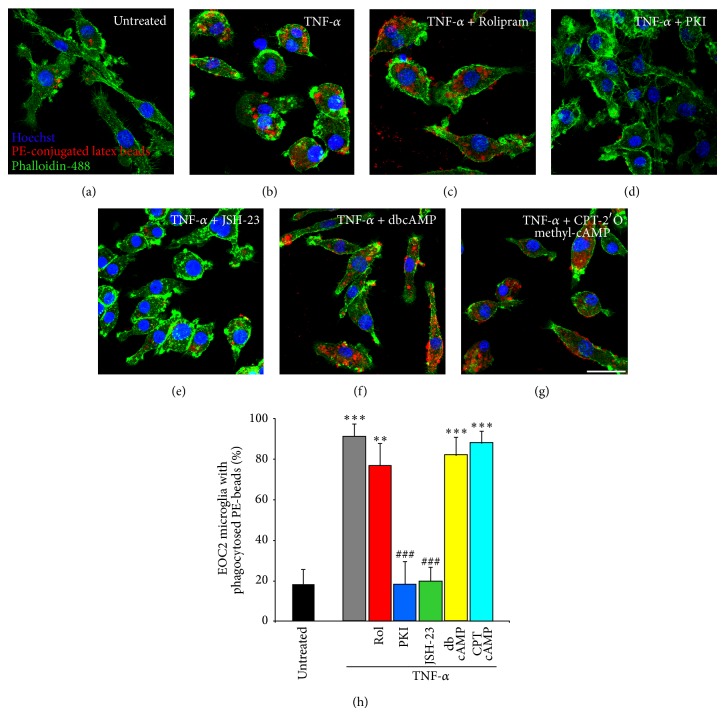
NF-*κ*B and PKA are important for TNF-*α*-induced increases in microglial cell phagocytic function. Phagocytic uptake of phycoerythrin-conjugated latex beads (red) is low in resting cells (a), though it is dramatically increased upon microglial cell activation with TNF-*α* (b). Elevating cyclic AMP levels in TNF-*α* stimulated EOC2 microglial cells by inhibiting PDE4 activity with Rolipram (c) or adding either PKA-specific cyclic AMP analog, db-cyclic AMP (f), or an EPAC-specific cyclic AMP analog, CPT-2′methyl cyclic AMP (g) does not reduce phagocytic ability. Conversely, antagonism of PKA with m-PKI peptide (d) or inhibition of p65 nuclear translocation with JSH-23 (e) reduces phagocytic activity in TNF-*α* stimulated microglia to levels comparative to untreated controls. Scale bar = 20 *μ*m. Cell morphology is shown by staining with phalloidin-488 (green) and the nuclear dye Hoechst (blue). Quantification of the percent of EOC2 microglial cells showing phagocytic uptake of PE-conjugated latex beads with different treatment conditions (h). The results shown are obtained from four independent culture plate replicates for each treatment condition. Errors are given as SEMs. Statistical significance to naive controls is indicated at ^**^ *P* < 0.01 or ^***^ *P* < 0.001 and versus 24 h post-TNF-*α* stimulation at ^###^ *P* < 0.001.

After identifying that multiple intracellular pathways are involved in the downregulation of cyclic AMP within microglia upon TNF-*α* stimulation and that cyclic AMP is critical for maintaining microglial cell homeostasis, largely through antagonizing NF-*κ*B signaling, we next wanted to examine how one of the main functions of microglia, phagocytosis, is affected by these manipulations. In these studies, EOC2 microglial cells were activated with TNF-*α* in the presence of phycoerythrin- (PE-) conjugated latex beads and inhibitors or activators of specific signaling pathways so as to examine their effects on microglial cell phagocytic function. For these experiments, 4 replicates were employed for each experimental condition and cells were fixed and analyzed at 24 hours after TNF-*α* challenge. In untreated EOC2 microglia, only a small fraction of the microglial cells phagocytosed the PE beads (18.1 ± 7.4%; Figure 7(a); black bar on Figure 7(h)), while TNF-*α* stimulation produced a significant increase in the ability of the cells to phagocytose PE beads (91.1 ± 7.4%, a 5-fold increase, *P* < 0.001; Figure 7(b); gray bar on Figure 7(h)). Cytokine-induced increases in phagocytic function were unaltered by the simultaneous addition of the PDE4 inhibitor Rolipram (10 *μ*M; 77.0 ± 10.6%; Figure 7(c); red bar on Figure 7(h)), the PKA activator db-cyclic AMP (1 mM; 82.2 ± 8.7%; Figure 7(f); yellow bar on Figure 7(h)), or the EPAC activator CPT-2′O methyl cyclic AMP (100 *μ*M; 88.2 ± 5.7%; Figure 7(c); aqua bar on Figure 7(h)). Conversely, TNF-*α*-induced increases in phagocytosis were inhibited to levels similar to that of untreated controls when there was concurrent application of the PKA inhibitor m-PKI (100 nM; 18.3 ± 11.2%; Figure 7(g); blue bar on Figure 7(h)) or the NF-*κ*B p65 nuclear translocation inhibitor JSH-23 (25 *μ*M; 19.4 ± 7.2%; Figure 7(e); green bar on Figure 7(h)).

## 4. Discussion

Stimulation of microglia with TNF-*α* leads to a rapid and persistent reduction in cyclic AMP that is accompanied by morphological and immunophenotypical changes as well as the secretion of various proinflammatory cytokines, reactive oxygen/nitrogen intermediates, proteases, and complement proteins, transforming microglia into phagocytic and antigen presenting cells [30]. These changes are accompanied by increases in PDE4 protein production and cyclic AMP-dependent PDE activity [21]. Previously we demonstrated that the inhibition of PDE4 with Rolipram, or more specifically* pde4b2* with RNA interference, in microglia could prevent the loss of cyclic AMP after activation with TNF-*α* [21]. In the current study, we examined the role of other signaling pathways in the negative regulation of cyclic AMP in microglia following TNF-*α* stimulation to better understand how crosstalk among signaling cascades regulates microglial cell activation during proinflammation phases of CNS injury or disease. In line with previous work, TNF-*α* stimulation of microglia led to significant increases in iNOS and COX-2 production [31] as well as higher cyclic AMP-dependent PDE activity [21], increased ERK1/2 phosphorylation [32], decreased I*κ*B [33, 34], increased NF-*κ*B phosphorylation at Ser-536 [34, 35], and its nuclear translocation [14, 36], which were accompanied by decreases in both cyclic AMP [21] and downstream Thr-197 phosphorylation of PKA [27]. Using specific pharmacological inhibitors, we identified that TNF-*α*-induced reductions in cyclic AMP in microglia could be completely abrogated through antagonism of NF-*κ*B, MEK, and PKA and partially reversed by PDE4 inhibition; we reported previously the role of PDE4 in cyclic AMP changes in microglia [21]. In further support of this role, antagonism of these pathways could also significantly prevent TNF-*α* induced increases in cyclic AMP-dependent PDE activity, suggesting that the signaling cascades investigated exhibited some degree of convergence upon the regulation of PDE activity directly or indirectly to affect intracellular levels of cyclic AMP. We also demonstrate that the rescue of cyclic AMP through targeting different signaling pathways can lead to disparate effects on activated microglia. These differences were evidenced in two different cell assays: the first, where the production of proinflammatory mediators COX-2 and iNOS in response to TNF-*α* in microglia was reduced by inhibitors of PDE4, PKA, MEK, and NF-*κ*B, and the second, a phagocytic functional assay, where TNF-*α* treated microglia retained their phagocytic function when cyclic AMP analogs (both PKA and EPAC specific) were used, but not when the NF-*κ*B nuclear translocation inhibitor, JSH-23, or the PKA inhibitor, m-PKI, were employed.

NF-*κ*B p65 phosphorylation at Ser-536, its untethering from I*κ*B, and subsequent nuclear translocation are critical for the induction of the proinflammatory gene expression program in microglia in response to the binding of ligands, such as TNF-*α*, to a diverse array of innate immunoreceptors, particularly TNFR1, IL-1R, and TLRs [37]. In our current work, TNF-*α* stimulation of EOC2 microglia induced this canonical NF-*κ*B signaling pathway, which was accompanied by a dramatic reduction in cyclic AMP and concurrent increase in cyclic AMP-dependent PDE activity. A direct link between the NF-*κ*B and cyclic AMP pathways was established through the finding that the p65 nuclear translocation inhibitor, JSH-23 [38], prevented TNF-*α*-induced decreases in cyclic AMP. JSH-23 is an aromatic diamine 4-methyl-N^1^-(3-phenyl-propyl)-benzene-1,2-diamine compound previously shown to inhibit LPS-induced DNA binding and nuclear translocation of NF-*κ*B p65, without affecting I*κ*B*α* degradation or its cytoplasmic recovery [38]. This may imply that cyclic AMP levels are regulated through NF-*κ*B via gene expression changes, particularly via the induced expression of* pde4* genes. Our data showed that there was a trend for a reduction in* pde4b2* mRNA upon inhibition of either PKA or NF-*κ*B at 3 hours after TNF-*α* stimulation, but this time point may have missed earlier and more significant changes in expression. Indeed, at 3 hours substantial reductions in cyclic AMP-dependent PDE activity were observed with PKA and NF-*κ*B inhibition that may have resulted from earlier antagonism of* pde4 *expression and protein production. Previously we have shown a robust expression of* pde4* mRNA in microglia within 1 hour of TNF-*α* or IL-1*β* stimulation [21]. Examination of the* pde4b* and* d* gene promoter regions has shown that they are rich in* CRE* and* AP-1* regulatory motifs [39]. To better understand how NF-*κ*B may be altering* pde4* gene transcription, further characterization of the* pde4* gene promoters through mutational analysis should be performed to examine the role NF-*κ*B activation plays in their expression.

Levels of cyclic AMP were also restored following m-PKI inhibition of PKA. While the catalytic subunits of PKA (PKAcat) play an important downstream role in communicating increases in cyclic AMP at the level of gene transcription, through phosphorylation and activation of the constitutive transcription factor CREB [40], it has been shown that another pool of PKAcat resides constitutively tethered to I*κ*B:NF-*κ*B p65 complexes in the cytoplasm [41, 42]. This pool of PKAcat is not bound by a regulatory PKA subunit, though it remains in the I*κ*B:p65 complex until I*κ*B*α* is phosphorylated and degraded [41]. At that time, PKAcat is released to phosphorylate NF-*κ*B p65 at Ser-276, a critical event that allows, upon nuclear translocation, the interaction of NF-*κ*B p65 with the transcriptional coactivator CBP/p300. The interaction between p65^Ser-276^ and CBP/p300 at the DNA displaces histone deacetylases (HDACs), such as HDAC1 and 3, which are associated with unphosphorylated nuclear NF-*κ*B proteins, allowing for the expression of NF-*κ*B-regulated genes [42, 43]. We hypothesize that m-PKI inhibition of NF-*κ*B-associated PKAcat and the ensuing inhibition of NF-*κ*B-regulated gene expression, like the nuclear translocation inhibitor JSH-23, antagonize the expression of genes involved in the negative regulation of cyclic AMP, primarily PDE4s. However, m-PKI could also elicit a number of other effects through inhibition of PKA on cyclic AMP and NF-*κ*B signaling including the block of PKA phosphorylation and increased activity of PDE4 long forms [23, 44] and preventing PKA phosphorylation of CREB, thus decreasing* pde4* gene transcription, which is present in a negative feedback loop when cyclic AMP levels are high [39].

At 3 hours after TNF-*α* stimulation, we also saw a significant induction of ERK1/2 phosphorylation that occurred in the temporal context of reductions in both cyclic AMP and downstream phosphorylation of PKA. Stimulation of innate immunoreceptors, TNFR1, IL-1R, or toll-like receptors (TLRs), can activate the MAPK pathways, ERK, JNK, and p38 [45]. One main upstream effector of these MAPKs is the MAP 3-kinase, tumor progression locus-2 (TPL-2; [46]). Interestingly, in* tpl-2* deficient macrophages, activation of ERK, but not JNK or p38, is completely inhibited following stimulation of TNFR1, TLR2, or TLR4 receptors [47, 48]. Although not examined in this study, it is likely that the change in ERK phosphorylation observed after TNF-*α* stimulation in microglia is mediated by TPL-2. In normal macrophages, TPL-2 is complexed with NF-*κ*B1 p105 and A-20 binding inhibitor of NF-*κ*B1 (ABIN-2), which maintain the protein stability of TPL-2 but prevent its MEK kinase activity [49, 50]. Like other Rel proteins, p105 release from TPL-2 and its degradation are triggered by IKK phosphorylation, though IKK phosphorylation at Thr-290 on TPL-2 itself has also been suggested to induce TPL-2 release in a mechanism independent of p105 degradation [51, 52]. Phosphorylation at Ser-400 of TPL-2 by IKK2 has additionally been shown to be required for LPS stimulation of TPL-2 MEK kinase activity in macrophages through a mechanism independent from p105 dissociation [53, 54]. Another MAP 3-kinase that may be responsible for ERK phosphorylation after TNF-*α* stimulation is transforming growth factor *β*-activated kinase 1 (TAK1), which has been identified to be downstream of TNFR1, IL-1R, and TLRs 4 and 9 [55, 56], though TAK1 has been insufficiently studied in macrophages and microglia in relation to MAP kinase signaling and appears to be more important for JNK and p38, rather than ERK, activation [56]. Investigating the role different MAP 3-kinases play in TNF-*α*-induced reductions in cyclic AMP would provide greater understanding on the crosstalk between the MAPK, NF-*κ*B, and cyclic AMP signaling pathways in directing the activation of microglia in response to proinflammatory stimuli.

Long forms of PDE4s contain two upstream conserved regions 1 (UCR-1) and 2 (UCR-2); UCR-1 contains the PKA phosphorylation site and an ERK phosphorylation site is located on the C terminus [44, 57, 58]. PKA phosphorylation at Ser-54 of PDE4s is associated with an increase in PDE4 activity in PDE4 long forms, ERK phosphorylation of Ser-579 is inhibitory [23, 58]. The main PDE4 spliced variant that we identified to be involved in decreasing cyclic AMP in TNF-*α* stimulated microglia previously was PDE4B2 [21]. PDE4B2 is the short-form isozyme of PDE4B that contains a UCR-2, but not UCR-1, meaning that its activity is not regulated by PKA phosphorylation, and conversely ERK phosphorylation has been found to enhance its activity [59]. In the current study, we showed that inhibition of the MEK-ERK pathway using PD98059 was able to prevent TNF-*α*-induced reductions in cyclic AMP. We postulate that one of the targets of ERK in activated microglia is PDE4B2 (and potentially other PDE4 short forms, like PDE4A1, which are increased upon proinflammatory stimuli [21]), where ERK can increase its activity and thus lead to greater cyclic AMP hydrolysis. Indeed, following TNF-*α* stimulation, we have shown that cyclic AMP-dependent PDE activity is significantly increased during the same period of reduced cyclic AMP [21]. PDE4B shows inducible expression in numerous immune cell populations [57, 60], including human monocytes [61] upon exposure to immune activators, like LPS. The critical role of PDE4B in regulating inflammatory responses was demonstrated in studies by Jin and colleagues [62, 63] in which the production of TNF-*α* in response to LPS was significantly reduced in peripheral leukocytes and macrophages from PDE4B, but not PDE4D, null mice. Of the PDE4B spliced variants, PDE4B2 is the only form to exhibit elevated expression around blood vessels and parenchyma, within infiltrating T cells and macrophages/microglia, following the induction of experimental autoimmune encephalomyelitis (EAE; [64]) and was increased in EOC2 microglia upon stimulation with TNF-*α* or IL-1*β* [21].

**Figure 8:**
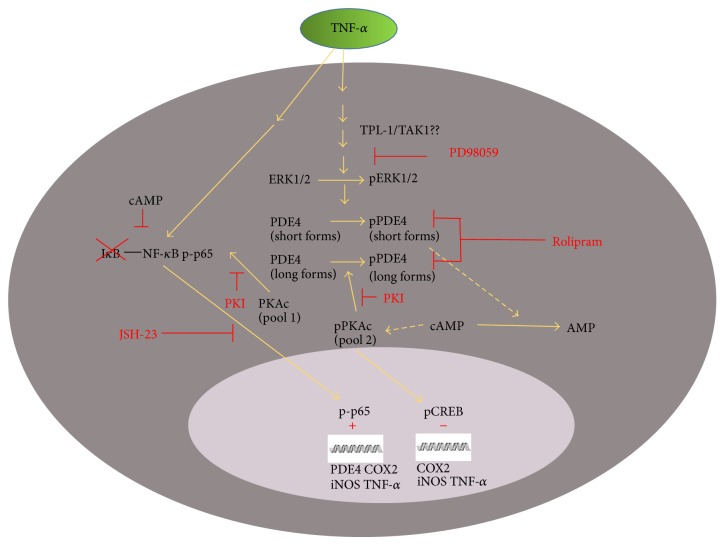
Cell signaling schema illustrating the proposed interactions between the PDE-PKA-cyclic AMP, MAPK, and NF-*κ*B pathways in regulating proinflammatory microglial cell phenotype and function in response to TNF-*α*. This cell diagram shows the proposed interactions between PDE-PKA-cyclic AMP, MAPK, and NF-*κ*B pathways in the cytoplasm and nucleus of microglia in response to TNF-*α* stimulation. These interactions control the proinflammatory gene program that alters microglial cell phenotype and function.

Crosstalk between the NF-*κ*B and cyclic AMP pathways may be required upon immunoreceptor activation to relieve the inhibition cyclic AMP imposes upon the NF-*κ*B pathway, thereby disrupting cellular homeostasis and permitting microglia to undergo changes in cellular activation towards a proinflammatory phenotype. The cyclic AMP signaling cascade has been demonstrated to largely inhibit the NF-*κ*B pathway at multiple levels, from (1) increasing cytoplasmic levels of I*κ*B, (2) CREB-mediated transcription of the* i*κ*b* gene, (3) interfering with ubiquitination and/or proteasomal degradation of I*κ*B, (4) inducing the exchange of transactivating NF-*κ*B complexes from activating (p50–p65) to repressive (p50–p50) complexes or, (5) the sequestering of CBP/p300 from DNA-bound p65 complexes by CREB to prevent NF-*κ*B-regulated gene transcription, among others (Figure 8). Through increased PDE4 gene expression and activity, the NF-*κ*B and MAPK pathways may work in concert, after their activation by proinflammatory signals, to promote cyclic AMP hydrolysis and thus remove this molecular brake to allow the autofeedback loop of inflammatory gene expression to proceed unabated for classical microglial cell activation.

In sum, we have shown that there is crosstalk at multiple levels of the NF-*κ*B and cyclic AMP pathways during TNF-*α* stimulation of EOC2 microglia. While a significant body of literature has characterized the signaling intermediaries and their interactions within both the canonical and noncanonical NF-*κ*B pathways in response to proinflammatory stimuli during the last two decades, the interactions between NF-*κ*B and cyclic AMP cascades upon microglia and macrophage activation and function, particularly those functions involving interactions with other CNS cell types such as apoptotic cell removal, axon growth, and degeneration, remain poorly understood. The importance of cyclic AMP in maintaining the homeostasis of the cell and its largely negative influence on NF-*κ*B signaling suggest that removing its brake function on NF-*κ*B, via its hydrolysis, may be necessary to enable NF-*κ*B to stimulate the full “classical” activation of microglia and macrophages from a resting or naïve state to a proinflammatory phenotype.
